# La Charité

**Published:** 1859-03

**Authors:** 


La Charité.—MM. Andral and Rayer have resigned their positions as Physicians to this institution, which they had so long held with credit and honor.

